# Supporting recruitment into complex trials: An embedded qualitative process evaluation in the RADICAL trial of radiofrequency denervation for chronic low back pain

**DOI:** 10.1177/20494637261450519

**Published:** 2026-05-16

**Authors:** Cecily K. Palmer, Vikki Wylde, Cathy Price, Kate E. Ashton, Andrew J. Moore

**Affiliations:** 1 Musculoskeletal Research Unit, Translational Health Sciences, Bristol Medical School, 1980 University of Bristol, Bristol, UK; 2 Musculoskeletal Research Unit, Bristol Medical School, 1980 University of Bristol and NIHR Bristol Biomedical Research Centre, University Hospitals Bristol and Weston NHS Foundation Trust and University of Bristol, Bristol, UK; 3 Community Specialist Services, Hampshire and Isle of Wight Healthcare Foundation Trust, Southampton, UK; 4 Bristol Trials Centre, Bristol Medical School, 1980 University of Bristol, Bristol, UK

**Keywords:** lower back pain, radiofrequency denervation, clinical trial recruitment, recruitment challenges, embedded qualitative research, rapid qualitative research, qualitative process evaluation, recruitment support

## Abstract

**Aims:**

A qualitative process evaluation was embedded into the pilot phase of the RADICAL randomised controlled trial of radiofrequency denervation for chronic and moderate-severe low back pain. The aim was to rapidly understand recruitment processes and inform development of strategies to overcome recruitment challenges.

**Methods:**

Audio recording of recruitment consultations between recruiting staff and potential patient participants, and interviews with patients who had consented to participate in the RADICAL trial. We used rapid thematic analysis to identify what information was provided, understanding of trial processes and potential challenges to recruitment.

**Results:**

Recruitment staff needed more support to clearly and effectively explain trial participation. Specific challenges included communicating trial procedures, explaining treatment equipoise and randomisation, and navigating the complex recruitment pathway. These findings led to the development of support materials for recruiters, including a process diagram and targeted training sessions to optimise information provision and informed consent.

**Conclusion:**

Embedding qualitative process evaluations within an RCT pilot can generate rapid insights into communication challenges and recruitment barriers. These insights can inform the development of practical support materials and process refinements to enhance recruitment, potentially strengthening trial viability.

## Background

Low back pain (LBP) is a leading cause of disability worldwide, with significant socioeconomic and personal consequences.^1^ For moderate-severe localised LBP arising from the facet joints and periarticular structures supplied by the medial branches of the primary dorsal rami, the National Institute for Health and Care Excellence (NICE) recommends that patients can be offered a diagnostic medial branch block (MBB) with local anaesthetic, and patients who respond positively can undergo radiofrequency denervation (RFD).^2^ RFD is a minimally invasive outpatient procedure which aims to reduce pain by interrupting the pain signal from the medial branch nerves to the brain by severing the nerves. Approximately 13,000 RFDs of the lumbar facet joints are performed annually in the National Health Service (NHS), with an associated NHS cost of approximately £22 million per year.^3^ However, there is uncertainty regarding the effectiveness of RFD due to a lack of evidence from high-quality clinical trials.^4–7^ To address this, the radiofrequency denervation for low back pain (RADICAL) trial was funded through a National Institute for Health and Care Research (NIHR) Health Technology Assessment (HTA) commissioned call. The RADICAL trial is a multicentre, pragmatic, double-blind, parallel-group, placebo-controlled, superiority randomised controlled trial (RCT), with a pilot phase which included an embedded process evaluation designed to optimise trial procedures.^8^

Qualitative process evaluation is increasingly recognized as a critical component of trial methodology, providing valuable insights into the contextual and procedural factors that influence trial success.^9,10^ Embedding qualitative research within clinical trials has been shown to improve trial design and implementation by offering a deeper understanding of participant behaviours, motivations, and decision-making processes.^10–12^ By exploring stakeholder experiences, such evaluations can help identify barriers and facilitators to recruitment, retention, and intervention fidelity. This evidence is particularly important in complex interventions like RFD, where outcomes may be influenced by nuanced interactions between participants, recruiters, and the trial context.^13^

The process evaluation undertaken during the pilot phase of the RADICAL trial underscores the broader methodological imperative of integrating qualitative inquiry into clinical research. Specifically, this evaluation aimed to refine recruitment strategies, enhance the clarity and accessibility of trial information for participants, and address barriers to participation, thereby laying a stronger foundation for the success of the full trial.

## Methods

### The RADICAL trial context

RADICAL is a double-blind, parallel group, superiority randomised controlled trial to evaluate the clinical and cost-effectiveness of RFD for chronic moderate-severe LBP. Ethics approval was obtained from the London – Fulham Research Ethics Committee (21/LO/0471). Full details of the trial methods are available in the published trial protocol.^8^ The trial aimed to randomise 250 patients from approximately 20 National Health Service (NHS) pain and spinal clinics.

During the pilot phase, patients meeting initial eligibility criteria and listed for a Medial Branch Block (MBB) were identified from clinical lists and provided with information about the RADICAL trial, including a patient information leaflet and recruitment video. Interested patients were contacted to arrange a recruitment consultation in which the trial was discussed and informed consent provided if the patient was willing to participate. Patients were eligible to proceed with randomisation if they met all the post-consent eligibility criteria including a positive response to the MBB (defined as ≥60% pain relief at 3 h post procedure).

Participants were randomised in theatre using a 1:1 allocation ratio to RFD or placebo RFD. RFD technique followed best practice guidelines developed for the trial.^14^ Placebo RFD followed the same protocol, but the electrode tip temperature was not raised. Participants who did not experience a clinically meaningful improvement in pain at 3 months after randomisation (defined as <2 point improvement on a pain Numeric Rating Scale) were offered the alternative intervention to the one provided at the outset, without the original allocation being disclosed. The primary clinical outcome was pain severity, measured using a pain Numeric Rating Scale, at 3 months after randomisation. Secondary outcomes were assessed up to 2 years after randomisation and include disability, health-related quality-of-life, psychological distress, time to pain recovery, satisfaction, adverse events, work outcomes and healthcare utilisation.

### Process evaluation design

Within the internal pilot phase of this trial, a qualitative process evaluation informed by the approach developed by the QuinteT team,^11^ was embedded to examine trial recruiters’ and patients’ experiences of the recruitment process and trial information. The aim was to identify any challenges experienced by recruiters or patients during the communication of trial information and consent procedures. The findings would then be used to refine recruitment materials and consent processes and to develop support strategies for site staff and participants for the full trial. These refinements and strategies aimed to address any barriers to participation and to improve adherence to trial protocols. Such iterative improvements align with best practices for enhancing trial feasibility and acceptability, as well as improving the quality and reliability of trial outcomes.^15^ Process evaluation can be located in subtle realist philosophy which accepts that social phenomena exist independently of research(er) observations, but that research knowledge is mediated through the prism of researcher experience and interpretation.^16^

Informed by the approach and methods comprising the QuinteT recruitment intervention^11^ qualitative data collection was integrated into the trial screening and recruitment stages and included a proposed 20 audio-recorded ‘observational’ recruitment consultations; and qualitative interviews with 20 patients prior to randomisation; 15 clinicians (including site Principal Investigators); and 10 recruiters across trial sites.

### Recruitment

Site teams were provided with information about the design and purpose of the qualitative study at their Site Initiation Visit (SIV). Once screening and invitation of potential patient participants to the trial had commenced, the research team made email contact with site staff to reintroduce the qualitative study and to provide a staff participant information leaflet (PIL) and consent form. The research team invited further questions regarding participation and asked interested site staff to complete a consent form for audio-recording of recruitment consultations and participation in an interview.

Two Public and Patient Involvement (PPI) representatives with experience of lower back pain contributed to the development and content of the patient facing study information. Patients were provided with information about the qualitative study within the main RADICAL trial PIL. Prior to a recruitment consultation taking place (either in person or by telephone), patients were approached for consent for audio recording and a written consent process completed. Patient consent to participate in a qualitative interview was included as an optional clause on the RADICAL trial consent form which was completed during the recruitment consultation. The research team were made aware of patients who had consented to interview participation when their baseline clinical details were collected, at which point they were known to be eligible to participate in the trial but pre-randomisation.

### Data collection

#### Audio-recording of recruitment consultations

Staff participants who completed a consent form were contacted to organise provision of an encrypted Olympus D9000 audio recorder with supporting guidance for use and upload of consultation audio recordings to the trial database. Recruitment consultation audio files were downloaded from the trial database and saved on the University of Bristol secure network.

#### Staff interviews

Staff participants were contacted by email to arrange a single 30-min telephone interview with the study researcher (CP) once patients at the site had been randomised/received interventions in the trial. A topic guide was developed to ensure exploration of key topics including views about trial involvement and processes, provision of information to patients, randomisation and patient equipoise, and any perceived challenges.

#### Patient interviews

Patient participants were contacted by telephone or email to reintroduce the qualitative study and arrange a single 30-min telephone interview with the study researcher (CP, female, experienced qualitative social scientist and health services researcher, PhD). Due to the natural time delay since completion of the RADICAL trial consent form, and to ensure informed consent, a verbal consent discussion preceded the interview. A topic guide was used to ensure exploration of key topics including understanding of trial purpose, study treatments, randomisation and views about information provided. Interviews were audio-recorded, downloaded to the University of Bristol secure network and transcribed verbatim by an external agency.

### Data analysis

Data collection and analysis were conducted concurrently and rapidly to enable feedback to the Trial Management Group (TMG) and allow team development of improvements to study processes.

Audio-recorded recruitment consultations were analysed using rapid qualitative analysis informed by the CLIPQ methodology to facilitate the rapid generation of findings.^17^ The qualitative team developed an a priori framework in which core topics important for patient understanding of the trial and a position of informed consent were listed.^17,18^ The core topics included understanding of trial purpose, equipoise/treatment uncertainty, randomisation, the treatment interventions, blinded treatment allocation, follow-up processes, and the 3-month blinded reintervention option. Rapid analysis sought to evaluate and analyse the content and process of recruitment consultations by reference to these predetermined topics of relevance, to identify good practice and any challenges, and inform actions to improve recruitment processes for the ongoing trial. Thematic saturation was not an aim of analysis.

Each audio-recording was transcribed verbatim by CP and entered into the framework under the a priori topic areas. Topic areas were modified and the framework extended as unanticipated content was identified (e.g. questions/misconceptions, discussion about the information materials provided to patients, and completion of the consent form). Each recruitment consultation was therefore transcribed in its entirety into the framework under either a priori or inductively generated topic areas with no omissions. Researchers then entered their comments on the content and communication of topics captured within the recording; summarising challenges identified in the explanation/comprehension of topics, and interpretation of study information. Two recruitment consultations were co-analysed by AM male, experienced qualitative social scientist, PhD. CP and AM met to discuss findings and differences of interpretation (of which there were none) and develop potential improvement processes for feedback to the TMG.

Interview transcripts were quality checked and anonymised by the qualitative team. Rapid descriptive analysis informed by the CLIPQ methodology^17^ was undertaken to identify key aspects of patients’ recruitment experience, to understand patients’ interpretation of study information and key processes for example, treatment uncertainty and randomisation, and to inform improved communication about the study. Transcripts were not returned to either staff or patient participants for comment and participants were not approached to feedback on findings.

## Findings

### Participants

In total, 23 site staff across six sites were approached to participate in the qualitative study between July 2022 and February 2023. Four audio-recorded recruitment consultations of approx. 15 min duration were received from two sites; each of which ‘observed’ the site primary investigator and a patient participant. No semi-structured interviews were undertaken with site staff due to a lack of capacity. Three semi-structured interviews of approx. 20 min duration were completed with three available patients from two sites (2 male, one female). As experienced across non-COVID trials more widely, recovery of research capacity after the COVID-19 pandemic posed significant challenges to the recruitment of both site staff and patients.^19^ The first trial site opened to recruitment in July 2022; however, further sites did not open until the end of 2022. Additionally, once opened, sites experienced slower recruitment than anticipated. Another challenge was that the long waiting times to undergo MBB resulted in delays to when patients could be approached for interview. The opportunity to complete interviews with site staff was also impacted; interviews were planned to take place once recruiting staff had conducted recruitment consultations on which to reflect, and clinicians had delivered RFD/placebo treatment within the trial. In addition, the return of consent forms from site teams was slow as many site teams had limited capacity and were unable to prioritise this aspect of the study. Also as time progressed it became apparent through conversations with site teams that Primary Investigators, rather than research nurses, were leading recruitment consultations at several sites.

### Recruitment consultations

Rapid analysis of recruitment consultations identified that they focussed primarily on the completion of the trial screening and consent forms. A need for further support in providing clear, accurate and comprehensive information to patients about trial participation was identified, including why the study was being done; what patients could expect if they participated in the trial, and clearer explanations about the uncertainty of RFD effectiveness, randomisation, blinding, further eligibility tests and blinded reintervention. Patients mainly accepted the information passively, with only a few questions about the practical aspects (e.g. use of anaesthetic/location of RFD clinic). The following challenges with communication of key trial information were identified:

#### Explanation of treatment uncertainty and equipoise

The single example in which the uncertainty of RFD effectiveness was referenced within the recruitment consultation revealed that uncertainty of this commonly used intervention may be difficult and awkward to explain:

‘the idea essentially [is] to find out what we claim we do… works [nervous laughter] ok’ (Rec cons 3)

#### Explanation of treatment groups

A need for further support was identified in relation to language used to explain the difference between the RFD and RFD placebo treatment groups. Explanation of treatment groups used technical specialist language potentially inaccessible for lay-persons (e.g. ‘intervention arms’), or value-laden language, for example, ‘sham/proper treatment’. Value-laden terminology may have implied that RFD treatment is known to be of superior effectiveness to placebo RFD.

‘[we] place the needles in - in one arm heat it up to 80 degrees, in another arm don’t do anything’ (Rec cons 3)

‘if there’s a positive response to the diagnostic injection [yes], the radio frequency or the sham … will be done by me’ [Rec cons 4]

#### Varied language use

Both MBB and RFD interventions were described using a variety of terms, for example, MBB was described as ‘test injections’ and ‘the blocks’; RFD was described as ‘the injection’, ‘denervation’, ‘the proper treatment’, and ‘lesioning’. We identified a need to use language that matched that used in the PIL and other materials; specifically ‘medial branch block/injections of anaesthetic into the nerves’ and ‘denervation/placebo treatment’.

#### Description of randomisation and blinding

We identified a need for further support in relation to explanations of randomisation and treatment blinding. Randomisation was only explicitly mentioned in one recruitment consultation:

‘what we call randomisation, allocate you into one of those groups’ [Rec cons 2]

#### Three-month blinded reintervention

The offer of blinded reintervention at 3 months after randomisation was dependent on whether patients had experienced a clinically important reduction in pain severity. However in one instance a recruiter suggested that all trial participants would receive two treatment interventions:

‘you will get the treatment, either for the first time or the second time so it’s not that you will not get the treatment, but there is a possibility that you will not get the proper treatment the first time’ [Rec cons 2]

### Interviews with patient participants

#### Patient recruitment experiences

During one interview it became apparent that the participant was unaware that they had agreed to participate in the RADICAL trial. This was reported back to the lead clinician at the recruiting site to allow them to recontact the patient. Analysis of interview data from the other two participants identified positive views about their interactions with site team staff and with the trial information that had been presented to them. Participants were keen to support medical research to benefit future patients. Participants reported having read the PIL but neither had seen the patient information video.

#### Understanding of key study information and processes

Two participants indicated clear understanding that the trial involved two treatments, and that the active RFD procedure involved use of heat to denature nerve endings.

‘they’re trying to see if it makes a difference, if the needle’s heated up when they’re using them, um, or vice versa’ [Patient participant 0004]

‘but it’s going to be almost like, like, like the burning of, of the, the nerve endings in the areas that’s, that’s affected [mhmm]. Erm, that – in, in a nutshell, without getting too technical,– that’s my understanding and what’s going to happen’ [Patient participant 0001]

Both participants had good understanding that treatment allocation would involve a random process, with one participant describing randomisation as completely acceptable to them.

‘[How do you feel about being randomly put into either the treatment group with the heated needles, or the treatment group with the unheated needles?]

It makes no difference to me at all’ [Patient participant 0004]

One interview participant however (mis)interpreted the appointment to have the MBB as an assurance that they would receive the active treatment in the trial. When the participant became aware within the interview that randomisation would take place after MBB, they were not comfortable with the possibility of receiving the placebo treatment and described a strong preference for the RFD treatment which they believed would be ‘doing something’ in comparison with a placebo treatment that ‘does nothing’, suggesting they were not in equipoise.

‘[How do you feel about being put sort of ‘at potluck’ into one or other of those treatments?] I just want something [yeah] as opposed to not if that makes sense [yes]. It was like it’s, it’s playing mind games with you, you know […] I was under the impression that I was just going to get, you know, not the placebo. I thought it was er, going to be the, the actual er, denervation…’ [Patient participant 0001]

This participant subsequently chose not to take part in the trial.

One participant recalled being informed about the 3-month blinded reintervention option, but another reported they were unaware of this element of the trial.

‘he said that, obviously, depending on which one I got, someone would be in touch with me and if it hadn’t, you know, made any improvement, I would get the chance to get the opposite procedure done.’ [Patient participant 0004]

‘[Did they explain about the three-month re-treatment option?] No…That doesn’t ring a bell either’ [Patient participant 0001]

For one participant the follow-up process involved in the trial was an appealing ‘bonus’ to participation:

‘he also said that the study, I’d be sort of not personally looked after, but someone would always be in touch with me at certain intervals…It was either a year or two years, sorry, that at certain intervals, they would be in touch with me’ [Patient participant 0004]

### Development of tailored support resources

#### Informed consent process diagram

Following presentation of early findings from the rapid qualitative analysis of recruitment consultations to the TMG, the RADICAL team collaboratively developed an ‘Informed Consent Process Diagram’ to support recruiters to provide optimal information to allow patients to make an informed choice about participation in the RADICAL trial (See Supplemental file 1). The process diagram presents key information topics in a suggested order for discussion with a patient during the recruitment consultation (and other conversations with patients), and suggested wording to support explanation of each aspect of the trial. Recommendations for neutral language and phrasing are provided to support avoidance of value-laden language (e.g. avoid using terms such as ‘the “real”/“fake” RFD treatment’). The Informed Consent Process Diagram was developed to respond to the specific support needs identified from our rapid analysis of the RADICAL recruitment consultations, whilst also being informed by the wider empirical literature on optimising informed consent and recruitment into trials.^11,12,20–24^ The Informed Consent Process Diagram was provided to all site teams in early January 2023.

#### Frequently asked questions document (FAQ)

In response to reviewing the findings from the embedded process evaluation, the TMG suggested a ‘Frequently Asked Questions’ document be developed to support recruiting sites to respond to patients’ questions and/or preferences and provide clear information consistent with the PIL and wording used in the Informed Consent Process Diagram. Using feedback from our Patient and Public Involvement representatives, the qualitative team developed a FAQ document (see Supplemental file 2) which provided ‘guided answers’ to questions, misconceptions, or preferences that were deemed likely to arise in relation to the study information materials and/or participation in the RADICAL trial.

In the Spring of 2025, an online investigators’ meeting was held, with 27 site staff when the trial was in the main phase of recruitment. In a poll about the use of the resources, 84% of respondents reported that they used the resources ‘often’ or ‘sometimes’.

#### Recruitment and informed consent training sessions

The qualitative team developed a training session to support site teams to optimise recruitment and informed consent, and to provide a forum to reflect on challenges both specific to RADICAL and common to trial recruitment more generally. Three online training sessions were delivered in March 2023, attended by 17 staff members from seven sites (6 Principal Investigators and 11 research nurses and other research support staff). An audio-recorded version of the training was also produced to increase accessibility and ensure availability to new site teams joining the RADICAL trial. Regular ‘drop in’ sessions have also been taking place to allow site teams to share and discuss current recruitment and trial issues and gain support and advice from the RADICAL trial team.

The training sessions provided empirically informed guidance on recognising and addressing specific recruitment challenges including: the importance of presenting treatment uncertainty as the starting point for the RADICAL trial^11,20–22,24,25^; explaining randomisation by focussing on the key RCT principle of fair comparison between groups, avoiding analogies to ‘coin toss’ or ‘decisions made by computers’^26–30^; responding to patients’ preferences;^23^ and use of neutral language when presenting trial treatments and other trial elements.^24^ Training also offered advice on presenting RADICAL-specific elements including the follow-up processes involved, the 3-month blinded reintervention option, and a reminder about consent form completion. The session incorporated interactive exercises to encourage reflection by site teams about current practice, followed by discussion of best practice. Site teams reflected on their team position of equipoise, discussed their description and explanation of the RADICAL trial treatments (and their difference) to patients, and considered responses to patient preference statements for ‘active’ RFD. Feedback gained during the session was used to inform revision and development of future training sessions.

Attendees at the training sessions were engaged and appeared to enjoy the opportunity to discuss topics and experiences, and to hear from and share learning with other teams. Reflection on the importance of equipoise revealed Principal Investigators varied in the extent to which they ‘accepted’ the uncertain effectiveness of RFD. However, the importance of neutral presentation of the two study treatments to patients was accepted by all Principal Investigators. Research nurses were often more comfortable that they could present trial treatments from a position of neutrality because many were from different specialties and RFD was not in their particular area of expertise. Most teams described the two trial treatments in terms of heated or non-heated needles consistent with the information provided in the PIL although some referred to ‘sham’ treatment which may imply inferiority to patients. The value of the patient information video was also discussed and steps taken to make it easier for site teams to make this available to patients. Training sessions had the additional benefit of facilitating greater familiarisation between the research team and individuals at site teams and their particular roles and responsibilities relating to the RADICAL trial. They also revealed how the implementation of the study protocol and screening and recruitment pathway varied across sites.

Training sessions with site staff were key in helping us to identify that our original screening and recruitment processes (to screen and recruit patients prior to MBB and then randomise only those patients who had a positive response to MBB [60% or greater pain relief]) were complex, and the associated workload a barrier, which limited capacity to recruit patients. The trial was designed this way to optimise acceptability to patients, introducing the trial before they were on an established pathway to RFD. However, the feedback from sites was that patients were willing to participate; motivated by the desire to help future patients, and reassured by the offer of blinded reintervention with the alternative treatment if they did not experience a clinically important improvement in pain after 3 months. To mitigate the risk to trial recruitment the TMG made a substantial protocol amendment to simplify the screening and recruitment processes by recruiting patients after they were listed for RFD. This approach substantially reduced the screening and recruitment workload to sites, reduced the time lag between consent and randomisation, and removed the need to consent many more patients than would be randomised. This amendment improved the trial randomisation rate (from an average of 0.1 patients/site/month pre-amendment to 0.3 patients/site/month post-amendment) and reduced the time between recruitment and randomisation (from an average of 4.8 months pre-amendment to 1.6 months post-amendment).

## Discussion

Despite the COVID-19-influenced delays in set-up that limited the scope of this embedded qualitative process evaluation, we provide a case-study which highlights how rapid analysis of even a small amount of qualitative data can be used to identify trial implementation and recruitment issues and develop solutions. This work, alongside insights gained from literature focussed on optimising recruitment into trials,^11,20,24^ underpinned the development of guidance and support materials for recruiters, and along with learning from this study, these may be beneficial to the wider research community.

It emerged that explanations of treatment uncertainty or equivalence can be particularly challenging in trials involving a comparison of an existing treatment with a placebo. Explaining that a currently used treatment has no evidence of effectiveness is likely to be more challenging for patients to accept, than if a trial compares two treatments of known effectiveness or an innovative treatment with a placebo. This resonates with the finding of Donovan et al. 2002^31^ that identified difficulties in staff presentation and patient acceptance of a ‘monitoring’ or ‘watch and wait’ intervention arm compared to the ‘radical treatment arm’ for prostate cancer. Previous studies have identified patient preference for the ‘active’ intervention and negative perceptions that an intention of placebo surgery is to ‘catch out the fakers’.^32^ In common with earlier studies, we also identified that clinicians may state a position of equipoise while simultaneously holding views about the ‘superiority of a particular treatment’,^22^ or experience discomfort when explaining uncertainty about treatment effectiveness.^12^ Some clinicians in our study appeared to vary in their acceptance of equivalence between the ‘active’ and placebo intervention.

Staff recruiting into trials are likely to benefit from support with communicating and presenting information about key study elements.^11,12,20,33^ Our analysis identified, in common with earlier studies, that presentation of treatment equivalence, as well as explanation of randomisation and blinding may be challenging for clinicians.^12,22,27^ Rapid identification of these challenges allowed the team to quickly respond by developing written guidance and training sessions, informed by the wider empirical research, to support site teams to mitigate them. Supporting trial-recruitment staff to provide clear and comprehensive information about all aspects of trial purpose and processes, using language that mirrors information provided in the trial PIL is likely to enhance recruitment and retention of participants. Studies show that the provision of training for recruiters in optimising communication about randomised trials enhances patient understanding and acceptance of randomisation.^33,34^ Realpe et al. (2016) highlight that early clarification of treatment uncertainty and equivalence provides a clear basis for explaining study purpose and randomisation.^21^ Guidance for use of neutral and factual language when describing trial interventions is important to avoid unintentional overvaluing of one trial intervention over another which may instil preferences amongst patients and undermine the key message of treatment equivalence.^35^ Use of the term ‘placebo’ should be carefully considered as the term may carry negative associations for some people.^32^

As a result of the learning from the embedded qualitative process evaluation and the subsequent development of training to reflect on challenges and support sites with recruitment we were able to implement a substantial protocol amendment to simplify the screening and recruitment pathway to recruit patients when they were post MBB and had been listed for RFD, thereby reducing the complexity and workload which impeded recruitment. Following the amendment, we observed improvement in randomisation rate and reduced time lag between recruitment and randomisation. This highlights the importance of embedded qualitative work within the pilot phase of a trial, and the need for proactive, rapid and appropriate actions to mitigate against identified barriers and challenges to trial delivery.

It is important to consider the limitations of this research when interpreting the results. Audio-recorded recruitment consultations gave an important but potentially incomplete insight into communication and information provision about the trial. Within the RADICAL screening and recruitment pathway recruiting clinicians may have completed an ‘initial conversation’ to gauge patients’ interest in the study prior to the audio-recorded consultation taking place. Recruiters may have provided fuller or clarifying information and checked comprehension of key study aspects at this earlier point in the pathway; this reflects the identification of recruitment as a protracted process taking place over different time points.^36^ While it could be considered a limitation that the data collected comprised three patient interviews and four consultation recordings (due to delays with trial set-up and progress), our study in fact demonstrates the quality of insight gained from even this modest quantity of qualitative data. Analysis was not aiming for theoretically informed, rich interpretations of data that might require thematic saturation, but instead to identify any immediate potential challenges, misunderstandings or misinterpretations of trial information during the recruitment process. This was to enable rapid practical changes rather than to generate theoretical understanding. Rapid, focussed analysis of the available interviews and consultation recordings enabled us to identify critical areas for improvement. We did not formally evaluate the Informed Consent Process Diagram or the training sessions provided for site teams, however evaluation of a complex intervention to increase randomisation and informed consent in the Prostate testing for cancer and Treatment [ProtecT] trial, which incorporated training and provision of support documents, identified that the rate of immediate acceptance of allocation had a continued rise following the delivery of the intervention.^11^

In conclusion, our recommendation is to include qualitative process evaluations within all randomised controlled trials to generate empirically-based insights to optimise communication and recruitment pathways. Rapid qualitative analysis supported by the use of an a priori framework can identify challenges in communication and understanding of key aspects of trial participation and determine where additional support or refinements to current processes are required. Early development of support resources such as an Informed Consent Process Diagram are likely to be beneficial, which can be updated to offer support for additional trial-specific challenges that are identified as the process evaluation progresses. Training sessions structured around a discussion of trial staff experiences and informed by evidence-based best practice can further support optimisation of communication and recruitment skills.

## Supplemental material

Supplemental material - Supporting recruitment into complex trials: An embedded qualitative process evaluation in the RADICAL trial of radiofrequency denervation for chronic low back pain Supplemental material for Supporting recruitment into complex trials: An embedded qualitative process evaluation in the RADICAL trial of radiofrequency denervation for chronic low back pain by Cecily K. Palmer, Vikki Wylde, Cathy Price, Kate E. Ashton and Andrew J. Moore in British Journal of Pain

Supplemental material - Supporting recruitment into complex trials: An embedded qualitative process evaluation in the RADICAL trial of radiofrequency denervation for chronic low back pain Supplemental material for Supporting recruitment into complex trials: An embedded qualitative process evaluation in the RADICAL trial of radiofrequency denervation for chronic low back pain by Cecily K. Palmer, Vikki Wylde, Cathy Price, Kate E. Ashton and Andrew J. Moore in British Journal of Pain

## Data Availability

Anonymised individual patient data will be made available for sharing after publication of the main results of the study from the University of Bristol Research Data Repository (https://data.bris.ac.uk/data/). Access to the data will be restricted to ensure that data is only made available to bona fide researchers for ethically approved research projects.
