# Supplementary material for: Supporting recruitment into complex trials: An embedded qualitative process evaluation in the RADICAL trial of radiofrequency denervation for chronic low back pain
Source: Br J Pain. 2026 May 16:20494637261450519. Online ahead of print. doi: 10.1177/20494637261450519 (PMC13179943; doi:10.1177/20494637261450519)
Supplement: Supplemental material - Supporting recruitment into complex trials: An embedded qualitative process evaluation in the RADICAL trial of radiofrequency denervation for chronic low back pain [file sj-pdf-1-bjp-10.1177_20494637261450519.pdf]

RADICAL Trial – Informed consent process diagram to support recruitment:

The RADICAL qualitative team have developed this process diagram to support recruiters during the recruitment consultation (and other parts of the screening /recruitment pathway) to provide full and clear information to allow the patient to make an informed choice about participation in the RADICAL trial. It is designed to be used in partnership with the 'Screening, consenting and enrolling working instructions 4a' (after step 7).

The diagram presents information topics and a suggested order for the information to be discussed with a patient during the recruitment consultation, with some example sentences for explaining the trial to patients. It also gives recommendations for **preferred and neutral language** when describing trial treatments and processes.

The process diagram can either be used to support the recruitment consultation in its entirety, or you may wish to refer to individual sections to guide the description and explanation of particular topics. In order for consent to be informed, all information topics included within the flowchart need to be discussed with a patient during the screening and recruitment process.

We expect to modify and further develop this resource as the trial progresses so please send any feedback to [email address]

RADICAL informed [illegible] recon[illegible]ended language and tips)

Acknowledge information already provided

- We sent you some information to invite you to participate in a research study (The RADICAL Study)
- I'd like to go through the information again to check that everything is clear and to answer any questions you may have.

> Please say 'research **study**' as it is preferred by patients to 'trial'

Give patient opportunity to share views

- Did you get a chance to read the information/watch the video about the study?
- What did you think of it?
- Do you have any questions?
- Do you think we could make anything clearer?

> Please pause and allow time for patients to share questions/ concerns.

What is RFD?

- The study is focussed on a treatment called Radiofrequency Denervation - do you feel that you are clear on what that involves?
- *If patient requires further info:* During radiofrequency denervation needles are placed next to the nerve of the painful facet joint(s) in the lower back, which are then heated up to cause a break in the nerve and hopefully stop the pain you are feeling. The treatment stops the nerve sending pain messages to the brain, and has no effect on the main nerves in the spine. The procedure is performed under local anaesthetic so you won't feel the heat. It takes place as a day procedure, in theatre.

Why is the study happening?

- Although this radiofrequency denervation is used in the NHS to treat certain types of back pain, there is actually uncertainty about how well it works for patients.
- At this hospital we are helping with a study that is working out how well the procedure works to reduce patients' lower back pain.
- If we can answer this question it will help clinicians like me when I am talking to patients such as yourself about treatment options for their back pain. Increasing our knowledge about whether or not this procedure works to reduce back pain is important for future patients.

Reassure patient

- Regardless of whether you take part in the study or not, you are going to continue to receive all the care and treatment we would normally provide for your back pain.
- Patients who take part in the study will not be expected to miss out on treatment that they would have had during the 'normal care' for this kind of back pain.

The study involves comparison of groups

- The best way to find out whether a treatment such as radiofrequency denervation works for patients is to compare two groups of patients.
- In this study, one group of patients will have the needle inserted into their back which will then transfer heat to the nerve (denervation).
- The other group of patients will have the needle inserted into their back but it will not transfer heat to the nerve (placebo treatment).

> Please say **'denervation / heated needles'** or **'placebo / unheated needles'** to match language in PIL. Do not use terms implying one treatment is 'better'. E.g. **'sham treatment', 'actual / real treatment'**

Treatment group is chosen by chance

- If you did decide to take part, the treatment group you are in will be decided by CHANCE – so whether you have the treatment with heated needles or the treatment with non-heated needles will be chosen at random.
- The reason for this is so that we can do a fair comparison between the two groups to find out whether one or other group had better pain relief.

> Please say **'treatment group'** (not **study arm / intervention group**)

Treatment group unknown to patient/ clinician team

- If you did decide to take part, you will also not be told whether you received the heated needle treatment or the unheated needle treatment, and the team here will not know which treatment you received.
- The reason for this is again to make sure that the two groups of patients can be fairly compared to find out which treatment is best for pain relief.
- If you wanted to know the treatment you'd had you would be told later on.

Three month retreatment option

- Once you have had the procedure you will be monitored by checking in with you regularly.
- After you receive treatment within the study, you will be contacted on 10 occa[illegible] years, to ask you some questions about your pain and how things are going. Th[illegible] the two treatments compare over the long-term.
- These follow-ups will be done over the telephone (5 calls over first 10 weeks) o[illegible] by post or online (5 questionnaires from 3 months to 2 yrs).

> Please say **'denervation/heated needles'** or **'placebo/unheated needles'** to match language used in the PIL.

Give patient opportunity to share views

Suggest asking one/two open questions:

- Is there anything I could explain further?
- Do you have any concerns?
- How would you feel about the treatment group you are in being decided by chance?
- How would you feel about being invited back for the alternative treatment if you don't feel you have had sufficient pain relief 3 months after the first treatment?

> Please pause and allow time for patients to share questions/ concerns.

Two further checks for suitability

- If you decide today that you're happy to take part in the study we would need to do two final checks to see if you're suitable to take part:
- The first involves you completing a questionnaire that asks questions about you, your pain and how you are feeling.
- The second is the Medial Branch Block – are you happy with what that involves?
- *If patient requires further info:* An MBB is when we do test injections of anaesthetic next to the nerve supplying the painful facet joints in the lower back, and we then ask you to complete some questions about your pain afterwards to see if the block works. The MBB gives us information about whether radiofrequency denervation is an appropriate treatment option for you.
- Once those two final checks have been done, we will be able to let you know whether you are suitable to take part in the study. If you are suitable, you'll then be told when to come in for your treatment.
- Even if you are not suitable for the study you can still get denervation as part of your normal care, and you will continue to be offered care for your back pain.

Give patient opportunity to share views

- Is there anything I could explain further?
- Is anything worrying you or is anything unclear?

SL2 & consent form

- Complete SL2 further eligibility criteria
- Proceed to consent form

Please ensure that all consent form clauses are read in full.
