# Supplementary material for: Supporting recruitment into complex trials: An embedded qualitative process evaluation in the RADICAL trial of radiofrequency denervation for chronic low back pain
Source: Br J Pain. 2026 May 16:20494637261450519. Online ahead of print. doi: 10.1177/20494637261450519 (PMC13179943; doi:10.1177/20494637261450519)
Supplement: Supplemental material - Supporting recruitment into complex trials: An embedded qualitative process evaluation in the RADICAL trial of radiofrequency denervation for chronic low back pain [file sj-pdf-2-bjp-10.1177_20494637261450519.pdf]

RADICAL Study - Frequently asked questions (FAQs) document

This document supports recruitment and informed consent by providing example patient questions/statements that may arise during discussions about participation in RADICAL. Suggested answers are provided to support informed consent, respond to patients' preferences or misconceptions, and provide clear information about study participation.

"I just want the pain to go away"

We are going to give you care and treatment for your pain regardless of whether you take part in the study. The reason we are doing the study is to find out for sure whether RFD does work well for people with pain like yours, which in turn will benefit future patients. Until we've done the study we won't know for sure whether RFD is better than placebo.

"Well I know I'll be chosen for the hot needles because my pain is so bad"

The strength of your pain is not a deciding factor in whether you get the RFD treatment or the placebo treatment. To ensure a fair comparison, that decision is made by chance. However you will receive care and treatment for your pain if you take part in the study and you will have the chance to receive both treatments if you feel the first did not work.

Although RFD is offered routinely, there is uncertainty about how well it works for people with pain like yours so we're asking people to take part in this study.

If you took part, you have an equal chance of getting either the RFD (in which the needles are heated) or the placebo treatment (in which the needles remain unheated). This would be chosen by chance. If you feel there has been no improvement in your pain three months later you would be invited back to have the alternative treatment to the one you had first time, meaning that you will have received both treatments, although you would not be told which one you had at each time point so as not to influence your views on whether it worked or not.

The study will allow us to know for sure whether RFD works well for patients, and can therefore benefit future patients.

"I just want the treatment that works now"

We are going to give you care and treatment for your pain regardless of whether you take part in the study.

Although RFD treatment is offered routinely, we are uncertain about how well it works for people with your kind of pain. This is why we are asking people to take part in the study. The study will allow us to find out for sure whether RFD works well for patients, and can benefit future patients.

If you took part, the treatment you'd receive – RFD (heated needle treatment) or the placebo treatment (in which needles remain unheated) - would be chosen by chance. If your pain was still bad three months later you would be invited back to have the opposite treatment to the one you had first time, meaning that you would receive treatment with both heated and unheated needles, although you would not be told which one you had at each time.

"Will there be anaesthetic?"

Yes. Whichever treatment you receive (RFD treatment, or the placebo treatment in which needles are unheated) will take place under a local anaesthetic in the operating theatre.

"How are you going to treat me if you aren't allowed to know what treatment I've had?"

The placement of the needle is the same in both treatments. The only difference is whether the needle is heated up or not. Once the needle is correctly placed – we use x-rays to help us do this very accurately - I will hand over to a team member who will operate the RFD machine to either heat up the needle or to leave the needle unheated, depending on which treatment group has been chosen for you. That team member will be the only person who knows on the day what treatment you have had, and it will be recorded in the study database so the research team at The University of Bristol can analyse the data. We do this so that no other people involved are influenced by what they think you had. At the end of the study the records will be made available and we can let you know which treatment you had if you wish.

"If I, the doctors and the research team don't know who has had the denervation or placebo, who does know?"

Once the needle is correctly placed – we use x-rays to help us do this very accurately - I will hand over to a team member who will operate the RFD machine to either heat up the needle or to leave the needle unheated, depending on which treatment group has been chosen for you. That team member will be the only person who knows on the day what treatment you have had, and it will be recorded in the study database so the research team at The University of Bristol can analyse the data. We do this so that no other people involved are influenced by what they think you had. At the end of the study the records will be made available and we can let you know which treatment you had if you wish.

"Could the placebo treatment help my pain?"

At the moment, we do not know whether RFD treatment is any more effective than the placebo treatment, and it is possible that either one of those treatment could help pain. The study will compare a group of patients that will have RFD with a group of patients that will have the placebo treatment in order to find out for certain whether RFD treatment is more effective than placebo treatment.

"I want to be treated with the heated needles so that my back pain is sorted as soon as possible"

We are going to give you care and treatment for your pain regardless of whether you take part in the study.

It is entirely up to you to decide whether or not you are willing to take part in the study but it's important that I explain some details before you make your final decision. RFD is offered routinely in the NHS, however, we're uncertain whether having RFD is effective for treating pain such as yours. This hospital is helping with the study because we are a research active hospital and this is a chance to find out for sure whether RFD works well for pain like yours.

If you took part in the study, you would be given either RFD treatment (in which needles are heated), or the placebo treatment (in which needles are unheated) and which one you would receive would be chosen by chance. We would then make contact with you at regular intervals to see how your pain was, and if your pain was still bad three months later you would be invited back to have the opposite treatment to the one you had first time, meaning that you would receive both treatments, although you would not be told which one you had at each time. You would not miss out on a treatment that you would
